# AP2a enhanced the osteogenic differentiation of mesenchymal stem cells by inhibiting the formation of YAP/RUNX2 complex and BARX1 transcription

**DOI:** 10.1111/cpr.12522

**Published:** 2018-11-15

**Authors:** Xiao Lin, Haoqing Yang, Lijun Wang, Wenzhi Li, Shu Diao, Juan Du, Songlin Wang, Rui Dong, Jun Li, Zhipeng Fan

**Affiliations:** ^1^ Laboratory of Molecular Signaling and Stem Cells Therapy, Beijing Key Laboratory for Tooth Regeneration and Function Reconstruction of Oral Tissues Capital Medical University School of Stomatology Beijing China; ^2^ Department of Implant Dentistry Capital Medical University School of Stomatology Beijing China; ^3^ Department of Endodontics Capital Medical University School of Stomatology Beijing China; ^4^ Department of Pediatrics Capital Medical University School of Stomatology Beijing China; ^5^ Molecular Laboratory for Gene Therapy and Tooth Regeneration, Beijing Key Laboratory for Tooth Regeneration and Function Reconstruction of Oral Tissues Capital Medical University School of Stomatology Beijing China; ^6^ Department of Biochemistry and Molecular Biology Capital Medical University School of Basic Medical Sciences Beijing China

**Keywords:** AP2a, BARX1, mesenchymal stem cells, osteogenic differentiation, yes‐associated protein

## Abstract

**Objectives:**

Bone regeneration by bone tissue engineering is a therapeutic option for bone defects. Improving the osteogenic differentiation of mesenchymal stem cells (MSCs) is essential for successful bone regeneration. We previously showed that AP2a enhances the osteogenic differentiation in MSCs. The present study investigated the mechanism of how AP2a regulates the direct differentiation.

**Materials and methods:**

Co‐immunoprecipitation and ChIP assays were carried out to investigate the underlying mechanism in MSCs differentiation. The osteogenic differentiation potential was determined by mineralization ability and the expression of osteogenic marker in vitro and the in vivo bone‐like tissue generation in nude mice.

**Results:**

We show that AP2a can compete with RUNX2, a key transcription factor in osteogenic differentiation, to recruit YAP and release the inhibition of RUNX2 activity from YAP by forming YAP‐AP2a protein complex. YAP‐AP2a protein complex also interacts with the *BARX1* promoter through AP2a, inhibit the transcription of BARX1. Moreover, BARX1 inhibits osteogenic differentiation of MSCs.

**Conclusions:**

Our discoveries revealed that AP2a may regulate the osteogenic differentiation in an indirect way through competing with RUNX2 to relieve the RUNX2 activity which inhibited by YAP, and also in a direct way via targeting the BARX1 and directly repressed its transcription. Thus, our discoveries shed new light on the mechanism of direct differentiation of MSCs and provide candidate targets for improving the osteogenic differentiation and enhancing bone tissue regeneration.

## 1 INTRODUCTION

As the physical support of a man, bone quantity and quality mean a lot to life and health. Numerous bone diseases are about to injure the normal bone structures. Through a century of developing, the surgical resection is still the main treatment to deal with bone tumours, including some cysts, leaving varisized bone defects.^1^, ^2^, ^3^ The surgeons are devoted to restore the supportive bone in a functional and aesthetic way, and achieve some progress with autografs, allografts and xenografts. However, the reconstruction of some large‐scale bone lesions, especially in maxillofacial areas, is inevitable with injuring other portion of the patients’ bone, such as the fibulas. And the outcomes are always unsatisfactory.^2^, ^3^ Moreover, bone fractures and osteoporosis usually happened to older patients. More than 30% of peoples over 50 year old are estimated to be attacked by osteoporosis‐related fracture.^4^ 5%‐10% of fractures may turn into non‐union or delayed union,^5^ of which the most severe one, hip fractures, are 15%‐25% consequent mortality.^6^ Along with the development of bone tissue engineering, tissue engineering strategies come to be an alternative to promote bone healing and the clinical treatment outcomes.

All the three portions, the seed cells, the scaffolds and the signalling molecules, in bone tissue engineering, are investigated widely and showed an enhancement of osteogenic differentiation or bone regeneration to a certain degree. The modification of the commonly used scaffolds, such as HA, β‐TCP, polylactic‐co‐glycolic acid (PLGA) and collagen, were carried out to improve the osteoinduction and osteoconduction.^7^, ^8^, ^9^ But the β‐TCP and porcine collagen were confirmed no better than bovine bone particles used in clinic.^10^ The signalling molecules BMPs and PRP have been widely used in clinic. But in larger bone defects, using growth factor alone is far from enough. Due to the tissue affinity, BMSCs are the most classic used seed cells for bone regeneration.^11^ In a comparison investigation, dental tissue‐derived MSCs were confirmed to have higher proliferation ability than BMSCs,^12^ which might be easier to amplify in vitro and gather enough cell quantity for clinic. Among the dental tissue‐derived MSCs, SCAPs exhibit the strongest osteogenic capacity compared with PDLSCs, DPSCs, SHED and DFSCs.^12^, ^13^, ^14^

It was well known that mesenchymal stem cells (MSCs) hold the ability to differentiate into osteogenic cells, and they are generally used as seed cells, the most important part, in bone tissue engineering. Learning about the mechanism of osteogenic differentiation in MSCs helps in improving the results of bone tissue engineering. Our previous study showed that activator protein 2a (AP2a) enhances the osteogenic differentiation potential of MSCs.^15^ AP2a is a transcription factor that can either activate or inhibit the expression of downstream gene through generating homodimer or heterodimer. It was confirmed to be an essential regulator in craniofacial development. The *AP2a* knockout mice developed severe skeletal defects with bone‐associated symptoms of craniofacial dismorphogenesis and duplicated limbs, forepaws or fingers.^16^, ^17^ The mutations of *AP2a* in human develop into the Branchio‐Oculo‐Facial Syndrome (BOFS) showing orofacial clefting similar with AP2a‐null mice.^18^, ^19^, ^20^ AP2a was also found to suppress chondrogenesis by downregulating the associated key transcription factor Sox5 and Sox6, as well as the matrix protein Col‐II and Col‐X.^21^ A most recent investigation found that the cooperation of AP2a and AP2b is the major regulator in neural crest development and affects the jaw skeleton patterning through the DLX code.^22^ All the studies showed the importance of AP2a in hard tissue differentiation and development. However, the mechanism of AP2a in osteogenic differentiation of MSCs remains unclear.

In the present study, we used dental and non‐dental‐derived MSCs to investigate the underlying mechanism of AP2a for directing osteogenic differentiation. Our results indicate that AP2a associates with Yes‐associated protein (YAP) to generate YAP‐AP2a protein complex. And RUNX2, a key transcription factor in osteogenic differentiation, can compete with AP2a to bind YAP and form YAP‐RUNX2 protein complex. Moreover, we found the directly downstream gene of AP2a, BARX1, which can inhibit osteogenic differentiation potential in MSCs. That means AP2a may regulate the osteogenic differentiation in an indirectly way through competing with RUNX2 to relieve the RUNX2 activity which inhibited by YAP, and also in a direct way via targeting the BARX1. This discovery is expected to promote the directed differentiation of MSCs for further applications in bone tissue regeneration.

## 2 MATERIALS AND METHODS

### 2.1 Cell culture

Tooth tissue acquisition complied with the approved guidelines established by Beijing Stomatological Hospital, Capital Medical University, with informed patient consent. The third molar was disinfected with 75% ethanol and then washed with phosphate‐buffered saline (PBS). We isolated and cultured the SCAPs, as described previously,^23^ and then identified the cell type. Briefly, SCAPs were separated from the apical papilla tissues. The tissues were separately digested in a solution containing 3 mg/mL collagenase type I (Worthington Biochem, Lakewood, NJ, USA) and 4 mg/mL dispase (Roche, Basel, Switzerland) for 1 hour at 37°C. Single‐cell suspensions were obtained by passing the cells through a 70‐μm strainer (Falcon; BD Labware, San Jose, CA, USA). WJCMSCs and BMSCs were purchased from ScienCell Research Laboratories (San Diego, CA, USA). MSCs were cultured in complete medium containing MEM alpha‐modified Eagle's medium (Invitrogen, Carlsbad, CA, USA), 15% foetal bovine serum (FBS; Invitrogen), 2 mmol/L glutamine, 100 U/mL penicillin and 100 µg/mL streptomycin (Invitrogen). The medium was replaced every 3 days. The cultured MSCs were placed in a humidified 5% CO_2_ incubator at 37°C. Human embryonic kidney 293 T cells were maintained in complete DMEM medium with 10% foetal bovine serum (FBS; Invitrogen).

### 2.2 Plasmid construction and viral infection

The plasmids were constructed using standard methods and verified by relevant restriction digestion and/or sequencing. Human full‐length BARX1 cDNA was fused to a haemagglutinin (HA) tag (HA‐BARX1) and subcloned into the pQCXIP retroviral vector with the AgeI and EcoRI restriction sites. Human full‐length AP2a cDNA was fused to a FLAG tag (Flag‐AP2a) and subcloned into the pQCXIP retroviral vector with the AgeI and PacI restriction sites. Human full‐length YAP cDNA was fused to a Myc tag (Myc‐YAP) and subcloned into the pQCXIH retroviral vector with the AgeI and EcoRI restriction sites. Human full‐length BCOR cDNA was fused to a FLAG tag (Flag‐BCOR) and subcloned into the pQCXIN retroviral vector with the AgeI and BamH1 restriction sites. The 2 kb upstream promoter of *BARX1* was subcloned into the pGL3‐Basic vector with the NheI and HindIII restriction sites to construct the luciferase reporter of the *BARX1* promoter. Short hairpin RNAs (shRNAs) containing the complementary sequences of the target genes were subcloned into the pLKO.1 lentiviral vector (Addgene, Cambridge, MA, USA) or LV3 lentiviral vector (Genepharma Company, Suzhou, China). A scrambled shRNA (Scramsh) was purchased from Addgene. The target sequences for the shRNAs are YAPsh (GCTTCAGGTCCTCTTCCTGAT), AP2ash (CGTTACCCTGCTCACATCA) and Consh (TTCTCCGAACGTGTCACGTTTC). For viral infection, MSCs were plated overnight prior to infection with retroviruses or lentiviruses in the presence of polybrene (6 µg/mL; Sigma‐Aldrich, St. Louis, MO, USA) for 6 hours. After 48 hours, infected cells were selected with different antibiotics for the appropriate periods.

### 2.3 Co‐immunoprecipitation assays

The cells were washed twice with PBS and lysed in 500 µL cold immunoprecipitation (IP) buffer (Pierce, Rockford, IL, USA) containing the complete protease inhibitor cocktail (Roche, Basel, Switzerland) for 15 minutes on ice. Lysates were centrifuged for 15 minutes at 4°C. The supernatants were collected and immunoprecipitated with the following antibodies: 2 µg rabbit polyclonal anti‐AP2a (cat no. sc‐184‐R; Santa Cruz Biotechnology, Santa Monica, CA, USA), 2 µg mouse monoclonal anti‐RUNX2 (clone no. C‐12; cat no. sc‐390715; Santa Cruz Biotechnology), or control normal rabbit IgG (cat no. sc‐2027; Santa Cruz Biotechnology) or mouse IgG (cat no. sc‐2025; Santa Cruz Biotechnology). After 1‐hour incubation at 4°C with gentle rotation, 40 µL of 50% protein A/G Plus‐Agarose slurry (Sigma, St. Louis, MO, USA) was added and the mixture incubated overnight at 4°C with gentle rotation. The immune complexes were collected by centrifugation and washed three times with 500 µl 20% cold elution buffer (cat no. 1858606; Thermo Scientific, Waltham, MA, USA). The immunoprecipitated proteins were detected by Western blot. The co‐immunoprecipitation assays related to the over‐expression construct with Myc tag was performed using the ProFound c‐Myc Tag IP/Co‐IP Application Set (cat no. 23622; Thermo Scientific) according to the manufacturer's guidelines.

### 2.4 Western blot analysis

RIPA buffer (10 mmol/L Tris‐HCL, 1 mmol/L EDTA, 1% sodium dodecyl sulphate [SDS], 1% NP‐40, 1:100 proteinase inhibitor cocktail, 50 mmol/L β‐glycerophosphate, 50 mmol/L sodium fluoride) was used to lyse the cells. The samples were separated on a 10% SDS polyacrylamide gel and transferred to polyvinylidenedifluoride (PVDF) membranes in a semi‐dry transfer system (Bio‐Rad, Hercules, CA, USA). The membranes were blocked with 5% dehydrated milk for 2 hours and then incubated with primary antibodies overnight. The immune complexes were incubated with horseradish peroxidase‐conjugated anti‐rabbit or anti‐mouse IgG (Promega, Madison, WI, USA) and visualized using SuperSignal reagents (Pierce). The primary antibodies were rabbit monoclonal anti‐BARX1 (clone no. EPR14120, cat no. ab181851, Abcam, Cambridge, UK), rabbit polyclonal anti‐AP2a (cat no. sc‐184‐R; Santa Cruz Biotechnology), rabbit monoclonal anti‐YAP1 (clone no. EP1674Y; cat no. ab52771; Abcam), mouse monoclonal anti‐RUNX2 (clone no. C‐12; cat no. sc‐390715; Santa Cruz Biotechnology), mouse monoclonal anti‐HA (clone no. C29F4; cat no. MMS‐101P; Covance, Princeton, NJ, USA), rabbit polyclonal anti‐Myc (cat no. sc‐789; Santa Cruz Biotechnology) and anti‐FLAG M2 (clone no. 9A3; cat no. 8146; Cell Signaling Technology, Beverly, MA, USA). The monoclonal primary antibody anti‐glyceraldehyde 3‐phosphate dehydrogenase (GAPDH; clone no. GAPDH‐71.1; cat no. G8795; Sigma‐Aldrich) was used to detect the housekeeping protein.

### 2.5 Alizarin red staining

Mesenchymal stem cells were grown in mineralization‐inducing medium using the STEMPRO Osteogenesis Differentiation Kit (Invitrogen). To detect mineralization potential, cells were induced for 2 or 3 weeks, fixed with 70% ethanol and stained with 2% Alizarin Red (Sigma‐Aldrich). To quantitate the calcium mineral content, the stained cells were destained with 10% cetylpyridinium chloride in 10 mmol/L sodium phosphate for 60 minutes at room temperature. The concentration was determined by the absorbance at 562 nm on a multiplate reader using a standard calcium curve in the same solution. The final calcium level in each group was normalized to the total protein concentrations obtained from a duplicate plate.

### 2.6 Reverse transcriptase polymerase chain reaction (RT‐PCR) and real‐time RT‐PCR

Total RNA was extracted from MSCs using Trizol reagent (Invitrogen). The cDNA was synthesized from 2 µg aliquots of RNA, random hexamers or oligo(dT), and reverse transcriptase according to the manufacturer's protocol (Invitrogen). Real‐time PCR reactions were carried out using the QuantiTect SYBR Green PCR kit (Qiagen, Hilden, Germany) and an Icycler iQ Multi‐colour Real‐time PCR Detection System. The primers for specific genes are listed in Table S1.

### 2.7 Luciferase assay

Human 293 T cells were plated 24 hours before transfection at a density of 2×10^5^ cells/well on a 12‐well plate. Co‐transfection was performed with 5 ng Renilla reniformis, 0.2 μg luciferase reporter *BARX1* promoter construct, 0.2 μg wild‐type Flag‐AP2a construct, 0.2 μg wild‐type myc‐YAP construct, or control vector (pQCXIP or pQCXIH) using the FuGENE 6 transfection reagent (Roche) in a 3:1 ratio (v/w) of reagent to DNA. Luciferase assays were performed using a dual‐luciferase reporter assay system (Promega) 48 hours after transfection. All reporter assays were normalized to Renilla.

### 2.8 ChIP assays

The ChIP assay kit (Merck Millipore, Billerica, MA, USA) was used according to the manufacturer's protocol. Briefly, 2 × 10^6^ cells were incubated with 1% formaldehyde for 10 minutes at 37°C. Polyclonal antibodies (2 µg) against AP2a (cat no. sc‐184‐R; Santa Cruz Biotechnology) were added to make DNA precipitate. Rabbit IgG (cat no. sc‐2027, Santa Cruz Biotechnology) was used as a negative control. All precipitated DNA samples were quantified by real‐time PCR. Quantification data were expressed as the percentage of input DNA. The real‐time PCR primers targeted the AP2a‐binding region of the *BARX1* promoter are listed in Table S2.

### 2.9 Transplantation in nude mice

The present study was censored and approved by the Animal Care and Use Committee of Capital Medical University, Beijing, China. Six female immunocompromised mice (BALB/C‐NU; 8 weeks old, 16‐18 g) were purchased from the Institute of Animal Science of Vital River Co., Ltd., Beijing, China. The mice were fully barrier‐reared with free access to water and a regular supply of food. Mixtures of approximately 4.0 × 10^6^ cells and 40 mg of hydroxyapatite/tricalcium phosphate (HA/TCP) ceramic particles (Engineering Research Center for Biomaterials, Sichuan University, China) were incubated at 37°C for 1 hour before being transplanted subcutaneously into the dorsal surface of the immunocompromised mice. All of the operations followed the regulations of the approved animal protocol. The transplants were harvested 8 weeks after surgery, fixed with 10% formalin, decalcified with buffered 10% EDTA (pH 8.0) and embedded in paraffin. After tissue slicing, the sections were deparaffinized, hydrated and stained with haematoxylin and eosin (H&E).

### 2.10 Immunohistochemistry staining

For immunohistochemistry staining, the hydrated tissue sections were incubated in 3% H_2_O_2_ for 10 minutes and then washed three times in PBS for 5 minutes. Epitope retrieval was performed by digestion with gastric enzyme for 20 minutes at 37°C and washed with PBS again. Ten per cent goat serum was used to incubate the sections for 15 minutes to block non‐specific antibody binding. After washing with PBS, tissue sections were incubated with a primary polyclonal antibody against BSP (cat No. ab52128; Abcam) overnight at 4°C. Tissue sections were transferred to 25°C for 30 minutes, then rinsed with PBS, incubated with horseradish peroxidase‐conjugated anti‐rabbit secondary antibody (Promega) at 25°C for 15 minutes, washed with PBS, incubated with detection reagents, counter‐stained with haematoxylin, dehydrated with gradient alcohol and mounted with neutral gum for light microscopy.

### 2.11 Statistical analysis

SPSS10 statistical software (IBM corporation, Amonk, NY, USA) was used in all statistical calculations. To determine significance, the Student's *t* test or one‐way ANOVA was performed and *P* ≤ 0.05 considered significant.

## 3 RESULTS

### 3.1 YAP associated with AP2a and formed protein complex in MSCs

**Figure 1:**
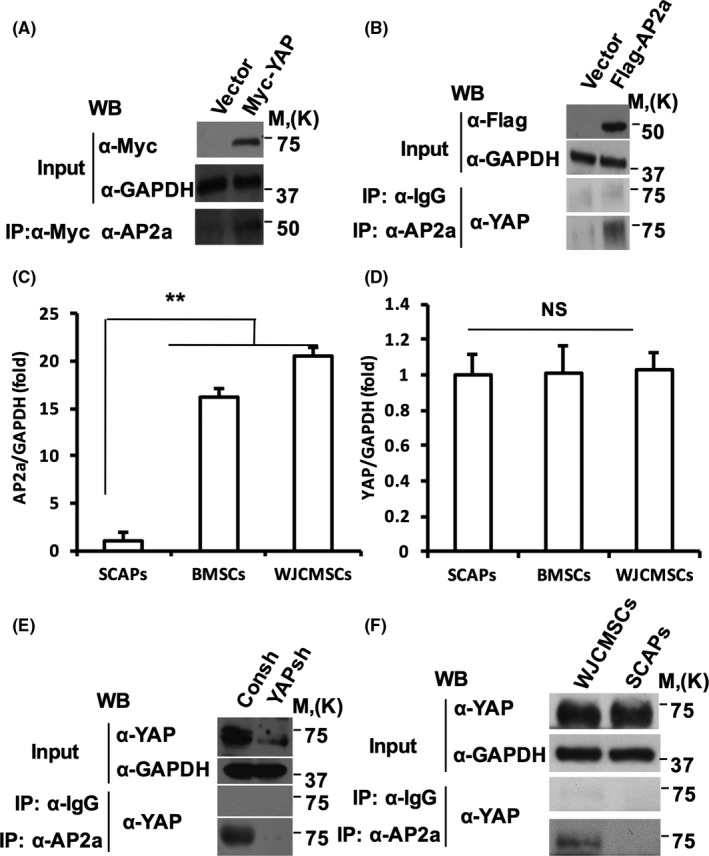
YAP associated with AP2a and formed protein complex in MSCs. A, Co‐IP results showed more AP2a‐YAP complexes formation in YAP over‐expressed SCAPs. B, Co‐IP results showed more AP2a‐YAP complexes formation in AP2a over‐expressed SCAPs. C and D, The expression of AP2a (C) and YAP (D) in SCAPs, WJCMSCs and BMSCs. GAPDH was used as an internal control. E, Co‐IP results showed less AP2a‐YAP complexes formation in YAP silenced WJCMSCs. F, Co‐IP results showed fewer endogenous AP2a‐YAP protein complexes in SCAPs than in WJCMSCs. One‐way ANOVA was performed to determine statistical significance. All error bars represent SD (n = 3). **P ≤ 0.01

We over‐expressed YAP in SCAPs using retrovirus expressing wild‐type YAP with a Myc tag. After selection with 400 μg/mL hygromycin for 10 days, Western blot result showed that YAP was ectopically expressed in SCAPs (Figure 1A). SCAPs were infected with retrovirus expressing wild‐type AP2a with a Flag tag and selected with 2 μg/mL puromycin for 7 days. Western blot result showed that AP2a was ectopically expressed in SCAPs (Figure 1B). Co‐IP results showed that more YAP‐AP2a protein complexes formed in SCAPs over‐expressing wild‐type Myc‐YAP or Flag‐AP2a (Figure 1A,B). We detected the expression of AP2a and YAP in SCAPs and non‐dental tissue‐derived MSCs including BMSCs and WJCMSCs. Real‐time RT‐PCR confirmed that the expression of AP2a was much lower in SCAPs than WJCMSCs and BMSCs (Figure 1C). However, there was no obvious difference in YAP expression between SCAPs, and WJCMSCs and BMSCs (Figure 1D). Then, YAP was knocked down in WJCMSCs by lentivirus infection. After selection with 2 μg/mL puromycin for 7 days, YAP was obviously knocked down by detected with Western blot. And the Co‐IP results showed that the depletion of YAP decreased the formation of YAP‐AP2a protein complexes in WJCMSCs (Figure 1E). Moreover, Co‐IP results showed fewer endogenous AP2a‐YAP protein complexes in SCAPs than in WJCMSCs, which further confirmed AP2a‐YAP protein complexes in MSCs (Figure 1F).

### 3.2 YAP inhibited the osteogenic differentiation of MSCs in vitro and in vivo

**Figure 2:**
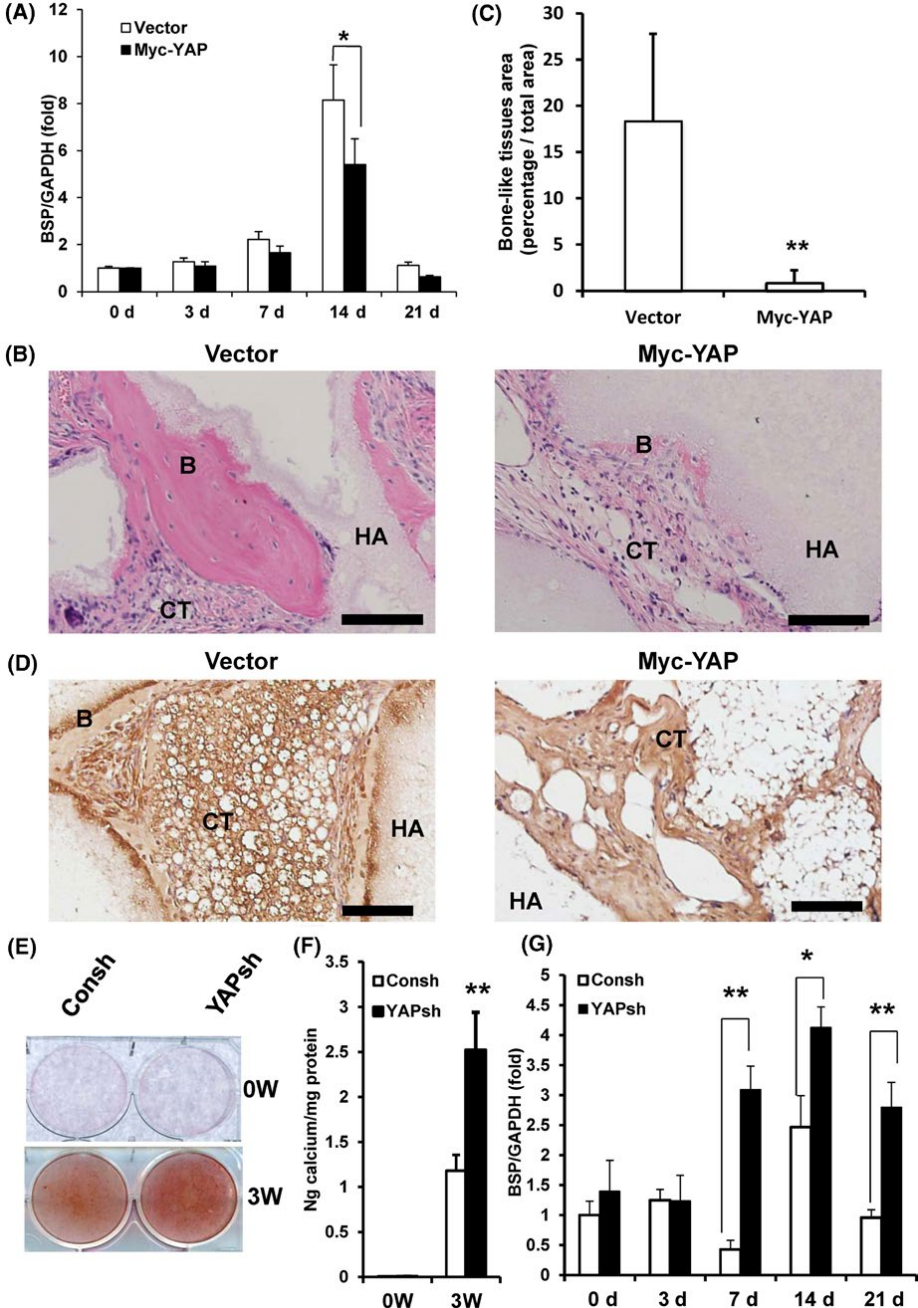
YAP repressed the osteogenic differentiation in MSCs in vitro and in vivo. A, Real‐time RT‐PCR results showed that the over‐expression of YAP decreased the expression of BSP in SCAPs. GAPDH was used as an internal control. The error bars represent SD (n = 3). B‐D, The over‐expression of wild‐type YAP reduced the bone‐like tissue formation in vivo. H&E staining micrographs showed the bone‐like tissue formation (B). The quantitative measurement of the bone‐like tissue. The error bars represent SD (n = 6) (C). Immunohistochemical staining micrographs to visualize distribution of BSP (D). Scale bar: 100 μm. E and F, Alizarin Red staining (E) and calcium quantitative analysis (F) in WJCMSCs. G, Real‐time RT‐PCR results showed that the knocking down YAP increased the expression of BSP in WJCMSCs. The error bars represent SD (n = 3). Student's *t* test was performed to determine statistical significance. *P ≤ 0.05. **P ≤ 0.01

The osteogenic differentiation potentials of SCAPs were examined after over‐expression of YAP. After culture with mineralization‐inducing medium, the Real‐time RT‐PCR results showed that over‐expression of YAP decreased the expression of BSP, an osteogenic marker, at 14 days after osteogenic induction in SCAPs compared to the vector group (Figure 2A). To investigate whether YAP expression affected the osteogenic potential of SCAPs in vivo, we transplanted SCAPs over‐expressing wild‐type YAP and the vector control subcutaneously into immunocompromised mice. Eight weeks later, the transplanted tissues were acquired and H&E staining results showed less bone‐like tissue formation in SCAPs over‐expressing YAP (0.83% ± 1.39%) compared to the vector groups (18.34% ± 9.46%) (Figure 2B,C). Furthermore, the immunohistochemistry staining results showed stronger BSP expression in vector groups than YAP over‐expressed SCAPs (Figure 2D). In addition, we also detected the osteogenic differentiation potentials of SCAPs after depletion of YAP in WJCMSCs. Alizarin Red staining and calcium quantitative analysis results revealed that knocking down of YAP enhanced the mineralization of WJCMSCs after induction (Figure 2E,F). Real‐time RT‐PCR showed that knocking down YAP promoted the expression of BSP at 7, 14, and 21 days after osteogenic induction in WJCMSCs (Figure 2G).

To determine whether YAP plays a similar role in the other MSCs, YAP was silenced in BMSCs by lentivirus infection. After selection with 2 μg/mL puromycin for 7 days, YAP was knocked down in BMSCs by real‐time RT‐PCR detection (Figure S1A). Alizarin Red staining results showed that silencing YAP in BMSCs enhanced mineralization after osteogenic induction (Figure S1B). Furthermore, knocking down of YAP also promoted the expression of BSP in BMSCs (Figure S1C).

### 3.3 AP2a competed with RUNX2 for binding with YAP

**Figure 3:**
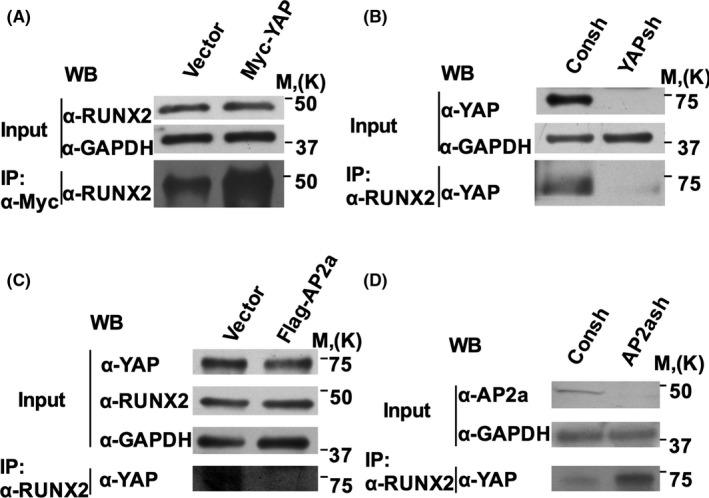
AP2a competed with RUNX2 for binding with YAP in MSCs. A‐D, Western blot showed individual signals (Input, 1% of lysate) and co‐immunoprecipitated protein complexes (IP, 99% of lysate). Co‐IP results showed more RUNX2‐YAP complexes in YAP over‐expressed SCAPs (A). Co‐IP results showed less RUNX2‐YAP complexes in YAP silenced WJCMSCs (B). Co‐IP results showed less RUNX2‐YAP complexes in AP2a over‐expressed SCAPs (C). Western blot showed more RUNX2‐YAP complexes in WJCMSCs silencing AP2a (D)

We examined the association of RUNX2 with YAP in SCAPs and WJCMSCs. Co‐IP results showed that the formation of YAP‐RUNX2 protein complexes was increased with Myc‐YAP over‐expression in SCAPs (Figure 3A). And depletion of YAP decreased the formation of YAP‐RUNX2 protein complexes in WJCMSCs (Figure 3B). In addition, Co‐IP results showed that the over‐expression of wild‐type Flag‐AP2a suppressed the association of YAP and RUNX2 in SCAPs (Figure 3C), and knocking down of AP2a in WJCMSCs promoted the association (Figure 3D). Taken together, these results indicate that AP2a may compete with RUNX2 for binding with YAP.

### 3.4 AP2a‐YAP protein complex directly inhibited the transcription of BARX1 in MSCs

**Figure 4:**
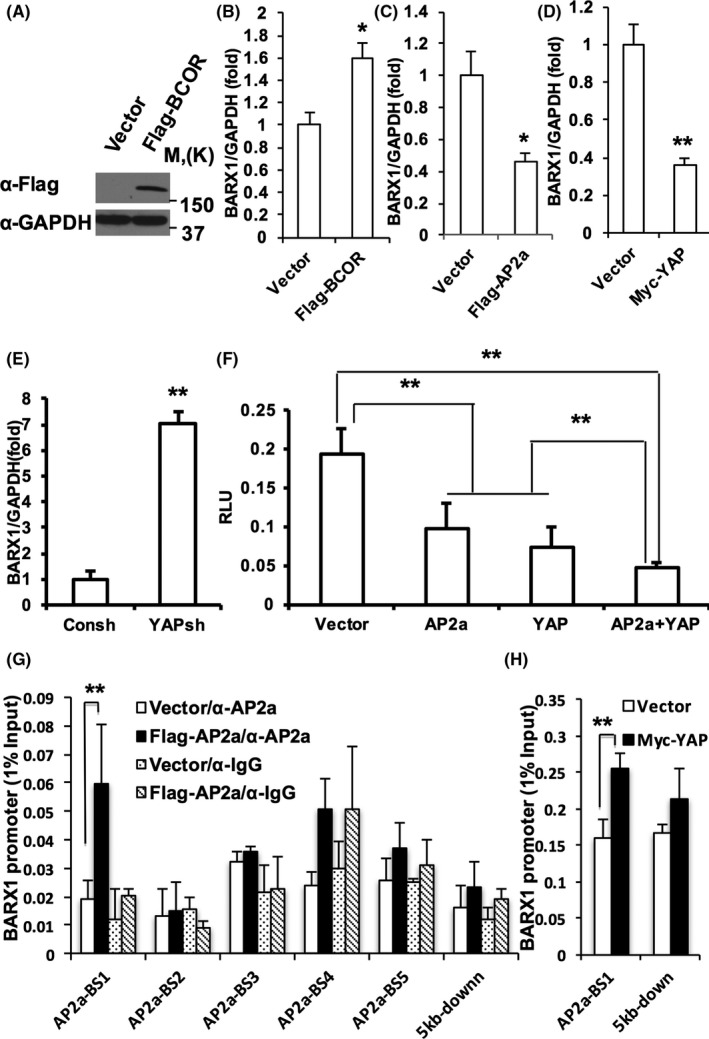
AP2a‐YAP protein complex directly inhibited the transcription of BARX1 in MSCs. A, Wild‐type BCOR was ectopically expressed in SCAPs as determined by Western blot. B‐E, Real‐time RT‐PCR results. Over‐expression of BCOR increased the expression of BARX1 in SCAPs (B). Over‐expression of AP2a repressed the expression of BARX1 in SCAPs (C). Over‐expression of YAP decreased the expression of BARX1 in SCAPs (D). Depletion of YAP increased the expression of BARX1 in WJCMSCs (E). GAPDH was used as an internal control. F, The luciferase assay results. G, ChIP assays showed Flag‐AP2a over‐expression enhanced recruitment of AP2a to *BARX1* promoter in SCAPs. H, ChIP assays showed Myc‐YAP over‐expression enhanced recruitment of AP2a to *BARX1* promoter in SCAPs. Student's *t* test (B‐E, H) or One‐way ANOVA (F, G) was performed to determine statistical significance. All error bars represent SD (n = 3). *P ≤ 0.05. **P ≤ 0.01

Next, we investigated the potential downstream gene of AP2a. We analysed the promoter of a transcription factor, *BARX1*, and uncovered five AP2a‐binding sites (Figure S2). And then, SCAPs were infected with retrovirus expressing wild‐type BCOR with a Flag tag. After selected with 600 μg/mL G418 for 14 days, Western blot results confirmed that BCOR was ectopically expressed in SCAPs (Figure 4A). Real‐time RT‐PCR results showed that the expression of BARX1 in SCAPs was increased by the over‐expression of BCOR compared to the vector group (Figure 4B). Moreover, real‐time RT‐PCR revealed that over‐expression of AP2a or YAP both repressed the expression of BARX1 in SCAPs at the mRNA level (Figure 4C,D). And knock‐down of YAP in WJCMSCs and BMSCs significantly increased the expression of BARX1 (Figure 4E, Figure S1D). Luciferase assay results revealed that over‐expression of AP2a and YAP inhibited the luciferase activity of *BARX1* promoter reporter (Figure 4F). ChIP assays results showed that more Flag‐AP2a proteins significantly associated with the one candidate AP2a‐binding site at* BARX1* promoter in AP2a over‐expressed SCAPs compared to the control group (Figure 4G, Figure S2), suggesting that AP2a directly promotes the transcription of BARX1. Furthermore, ChIP assays results also showed that more AP2a protein significantly associated with the* BARX1* promoter in YAP over‐expressed SCAPs compared to the control group (Figure 4H).

### 3.5 BARX1 repressed osteogenic differentiation of MSCs in vitro

**Figure 5:**
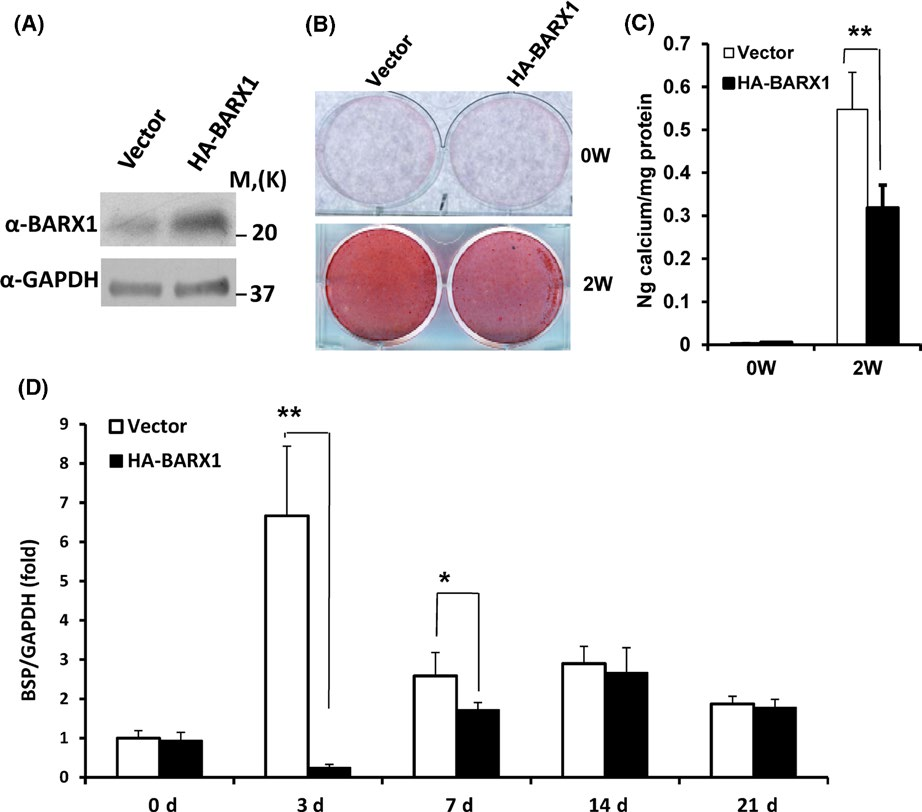
The over‐expression of BARX1 repressed osteogenic differentiation of SCAPs in vitro. A, Wild‐type BARX1 was ectopically expressed in SCAPs as determined by Western blot. B and C, Alizarin Red staining (B) and calcium quantitative analysis (C) showed that the over‐expression of BARX1 inhibited mineralization in SCAPs. D, Real‐time RT‐PCR results showed that BARX1 over‐expression decreased the expression of BSP in SCAPs. GAPDH was used as an internal control. Student's *t* test was performed to determine statistical significance. All error bars represent SD (n = 3). *P ≤ 0.05. **P ≤ 0.01

Next, we investigate the osteogenic differentiation potentials of SCAPs after over‐expression of BARX1. We over‐expressed wild‐type BARX1 in SCAPs via retrovirus infection. After selected with 2 μg/mL puromycin for 7 days, Western blot results showed that wild‐type BARX1 was ectopically expressed in SCAPs (Figure 5A). Alizarin Red staining and calcium quantitative analysis results showed that the over‐expression of BARX1 inhibited mineralization in SCAPs compared to the vector group after 2 weeks of induction (Figure 5B,C). We also examined the osteogenic marker BSP at the mRNA level. Real‐time RT‐PCR results showed that BARX1 over‐expression downregulated BSP at 3 and 7 days after osteogenic induction (Figure 5D). Taken together, these data demonstrate that BARX1 repressed the osteogenic differentiation of SCAPs in vitro.

To determine whether BARX1 possesses similar functions in other MSCs, BARX1 was over‐expressed in WJCMSCs and BMSCs by infected with retroviral constructs (Figures S3A and S4A). Alizarin Red staining and calcium quantitative analysis showed that the over‐expression of BARX1 inhibited mineralization of WJCMSCs compared to the vector group after 3 weeks of induction (Figure S3B,C). Real‐time RT‐PCR results showed that over‐expression of BARX1 downregulated BSP expression after osteogenic induction in WJCMSCs (Figure S3D). Similarly, real‐time RT‐PCR showed that over‐expression of BARX1 also downregulated BSP expression after osteogenic induction in BMSCs (Figure S4B).

## 4 DISCUSSION

Our previous study showed that AP2a enhances osteogenic differentiation in MSCs.^15^ To elucidate the underlying mechanism, we analysed the protein structure of transcription factor AP2a and found a PY motif, which is essential for transcriptional activation. Another protein module, the WW domain, could recognize and bind the PY motif to mediate protein‐protein interactions.^24^, ^25^, ^26^, ^27^ By protein structure analysis, we noticed that YAP had a WW domain. Using yeast two‐hybrid screening, YAP has been shown to associate with the PY motif of PEBP2a through its WW domain.^28^ Previously, YAP was confirmed to inhibit the osteogenic activity of osteoblasts.^29^, ^30^ Moreover, the physical stimulations, including hierarchical structure, acoustic tweezing cytometry, gold nanoparticles, grid topology, could enhance the osteogenesis through YAP activation.^31^, ^32^, ^33^, ^34^ All of these investigations confirmed that YAP plays an important role in osteogenic differentiation and bone formation. Therefore, we speculated that YAP may associate with the PY motif of AP2a through the WW domain and mediate its function. Indeed, the Co‐IP assays confirmed that AP2a can associate with YAP in MSCs. We further investigated the role of YAP for regulating the osteogenic differentiation of MSCs. Functional studies showed that YAP inhibits osteogenic differentiation in vitro and reduces bone‐like tissue generation in vivo. However, our previous study demonstrated that AP2a enhances osteogenic differentiation of MSCs, indicated that YAP is a transcription co‐repressor of AP2a and counters the function of AP2a. We further investigate how YAP‐AP2a complex regulates the osteogenic differentiation of MSCs. Previous investigations showed that YAP interacts with the Runx2 protein, a key osteogenic differentiation transcription factor, through the WW domain and PY motif in osteoblasts, and suppresses Runx2 activity, regulating skeletal gene expression and differentiation.^30^ While in present study, our results also confirmed that the WW domain of YAP interacts with the PY motif of RUNX2, generating YAP/RUNX2 protein complexes in MSCs. We speculate whether AP2a could compete with RUNX2 to associate with YAP. Indeed, by Co‐IP assays, we found that AP2a can compete with RUNX2 to bind YAP, more amount of AP2a protein in MSCs could reduce the RUNX2/YAP protein complex and diminish the inhibition of RUNX2 activity by YAP association in MSCs. That could be the reason why over‐expression of AP2a presented to enhance the osteogenic differentiation in MSCs. In the same time, by the protein structure analysis, AP2a and RUNX2 applied the binding function with the WW domain of YAP protein through their same PY domain, indicating that RUNX2 might also have the potential to compete with AP2a for binding YAP and diminished AP2a/YAP protein complex and its function.

Next, we want to figure out the target of AP2a/YAP protein complex in osteogenic differentiation regulation in MSCs. In our previous studies, AP2a is a direct target of BCOR and negatively regulated by BCOR via epigenetic regulation.^15^ And AP2a was reported to regulate some homeobox genes.^35^ Our previous microarray results showed that homeobox gene BARX1 is downregulated in BCOR mutant SCAPs compared to wild‐type SCAPs.^15^ As a transcription factor, *BARX1* expresses in the mesenchyme and plays a crucial role in craniofacial mesenchyme development.^36^, ^37^ In the mouse, the pharyngeal arches, limb buds, developing joints, molar tooth papillae and the stomach wall, where the mesenchymal condensation is located, exhibit significant Barx1 expression.^38^, ^39^, ^40^ Some joint and craniofacial anomalies in humans are also caused by rare duplications and deletions of *BARX1*.^41^, ^42^, ^43^ To determine whether BARX1 is the downstream gene of BCOR, BCOR was over‐expressed in SCAPs and the expression of BARX1 was detected. The results clarified that BARX1 expression was increased by over‐expression of BCOR. Then, we wondered that BARX1 was regulated by BCOR directly or indirectly. The promoter of BARX1 was analysed to investigate the potential relationship among BCOR, AP2a and BARX1 and revealed that five candidate AP2a‐binding sites in the *BARX1* promoter.^44^ These findings suggested that BARX1 may be regulated by AP2a directly. In present study, we found that the expression of BARX1 was decreased after over‐expressing AP2a in MSCs, and luciferase and ChIP assay results confirmed that AP2a downregulated the transcription of BARX1 by directly binding the BARX1 promoter, confirmed that AP2a is a direct regulator of *BARX1*. These results suggest that BARX1 might be an indirect target of BCOR, and that its expression is mediated by AP2a which controlled by BCOR.

While, the functional studies showed that BARX1 inhibited the mineralization and osteogenic differentiation marker, BSP in MSCs. These findings indicate that BARX1 is a negative regulator for osteogenic differentiation in MSCs. Our previous study discovered that BCOR negatively regulates the osteogenic differentiation, and AP2a positively regulates the osteogenic differentiation in MSCs.^15^ While in present study, BCOR upregulated the expression of BARX1, and AP2a downregulated the expression of BARX1. These results are consistent with our previous findings.

Moreover, by luciferase assay, as the partner of AP2a, YAP is found to enhance the inhibition of AP2a for the *BARX1*. And ChIP assay also showed that YAP prompts the recruitment of AP2a at its binding site in the *BARX1* promoter, confirming that YAP could help AP2a to target BARX1 promoter and the inhibition of AP2a for the BARX1 transcript need YAP assisting. Interestingly, our results proofed that YAP also negatively regulated the expression of BAXR1, and functional study revealed that YAP inhibited osteogenic differentiation in MSCs. In skeletal lineage cells, previous investigation found that depletion both YAP and TAZ damaged the bone structures, matrix quality and caused fractures in vivo, inhibit osteogenic and collagen‐related genes expression in vitro and in vivo.^45^ Moreover, in some conditions, YAP activation could enhance the osteogenesis.^31^, ^32^, ^33^, ^34^ These evidences indicated that YAP‐mediated function and mechanism for osteogenic differentiation are complicated and suggested that regulation of YAP expression might not be a suitable method for controlling the directed differentiation, and interrupting the YAP partner or its protein complex formation may be the better way.

## 5 CONCLUSION

In the present study, our discoveries revealed that YAP protein could associate with RUNX2 or AP2a to form different protein complexes separately, AP2a competed with RUNX2 to recruit YAP by same PY domain in MSCs. AP2a/YAP complex directly downregulate the transcript of BARX1, which is a negative regulator for osteogenic differentiation in MSCs. Taken together, our discoveries suggested that AP2a may regulate the osteogenic differentiation in an indirect way through competing with RUNX2 to relieve the RUNX2 activity which inhibited by YAP, and also in a direct way via inhibiting the BARX1 in MSCs. Thus, our discoveries shed new light to the mechanism of direct differentiation of MSCs and provide candidate targets for improving the osteogenic differentiation and enhancing bone tissue regeneration.

## CONFLICT OF INTEREST

None.
