# Supplementary figures and images for: AP2a enhanced the osteogenic differentiation of mesenchymal stem cells by inhibiting the formation of YAP/RUNX2 complex and BARX1 transcription
Source: Cell Prolif. 2018 Nov 15;52(1):e12522. doi: 10.1111/cpr.12522 (PMC6430486; doi:10.1111/cpr.12522)

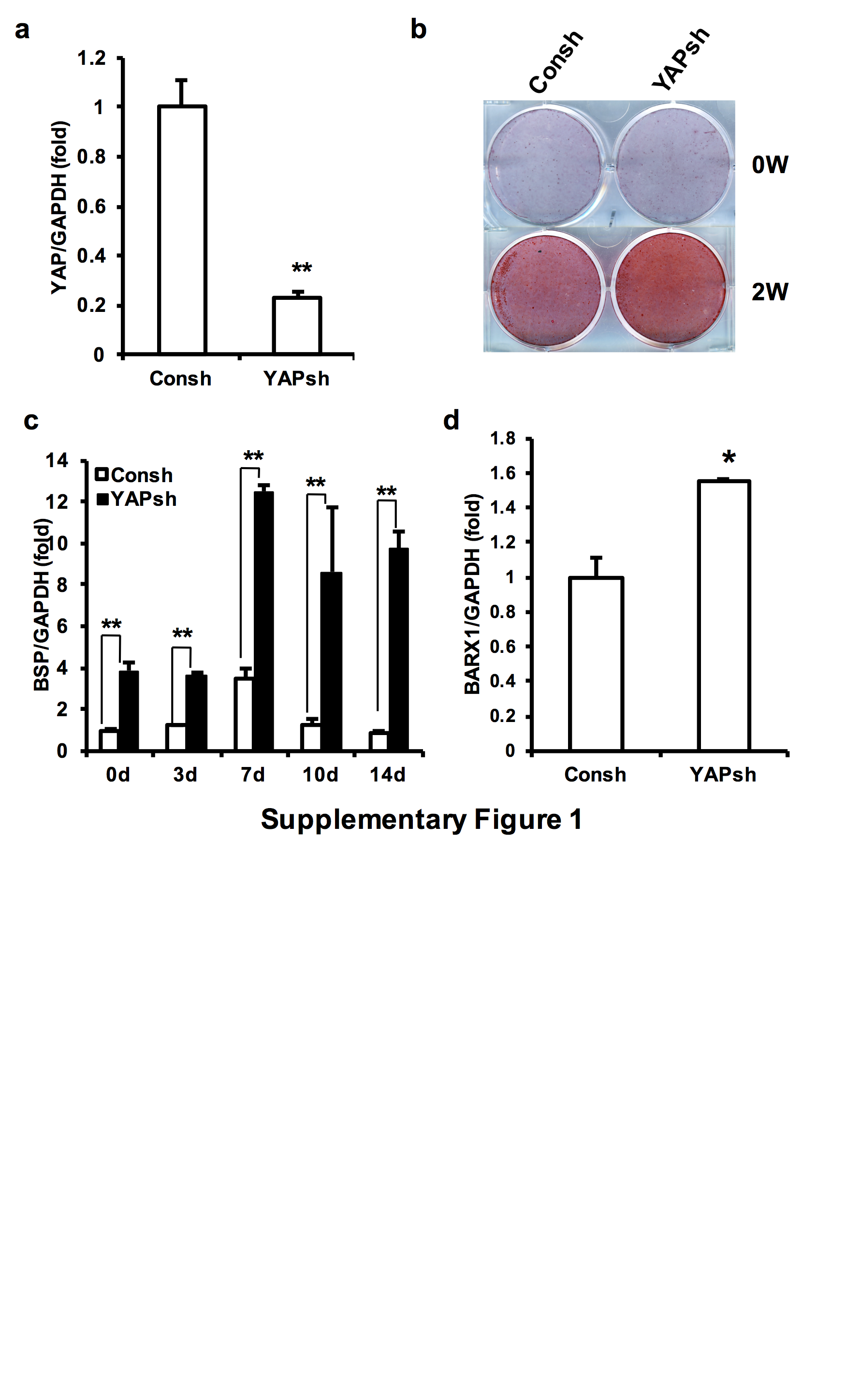

Supplement: Supplementary file 1 [file CPR-52-e12522-s001.tif]

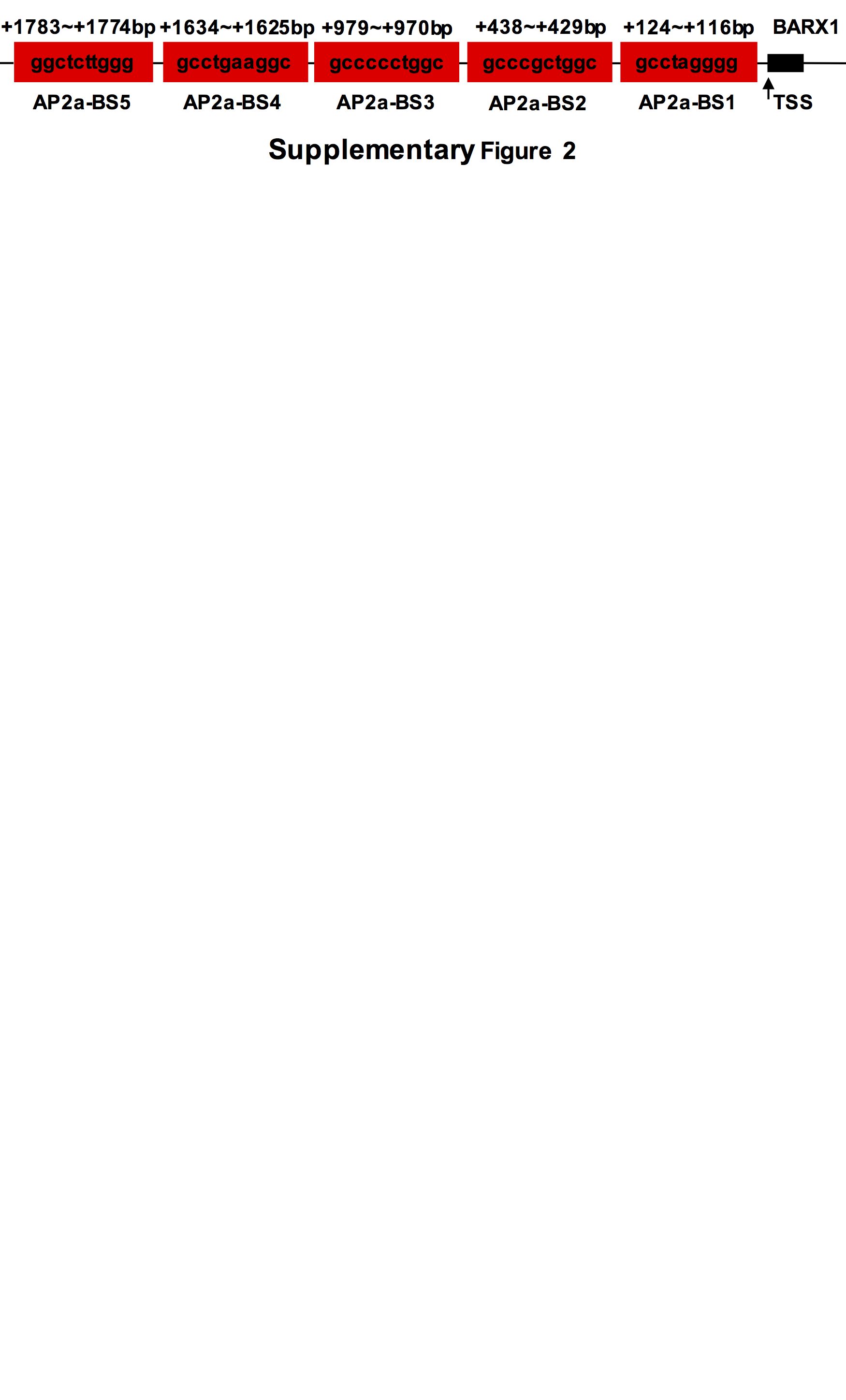

Supplement: Supplementary file 2 [file CPR-52-e12522-s002.tif]

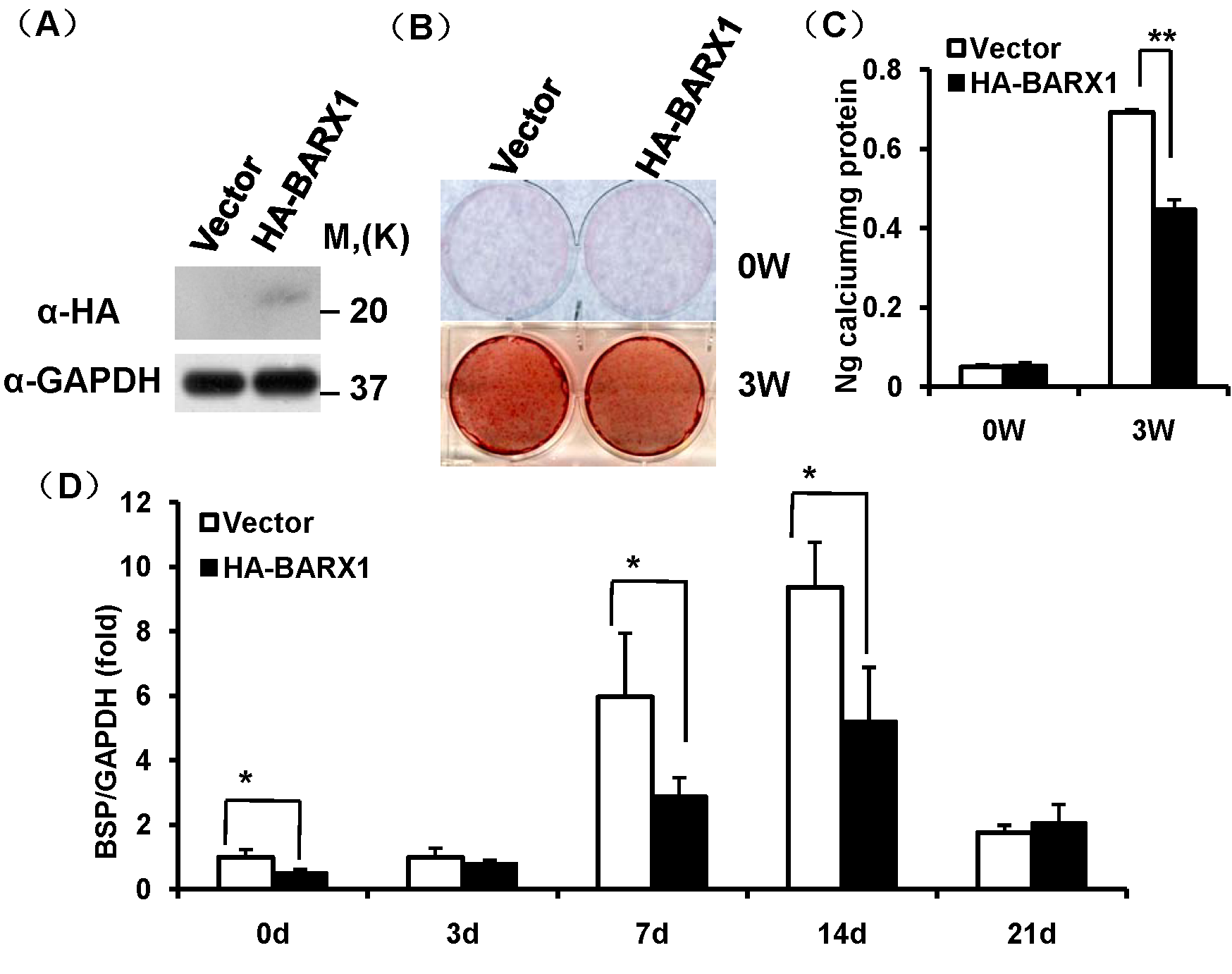

Supplement: Supplementary file 3 [file CPR-52-e12522-s003.tiff]

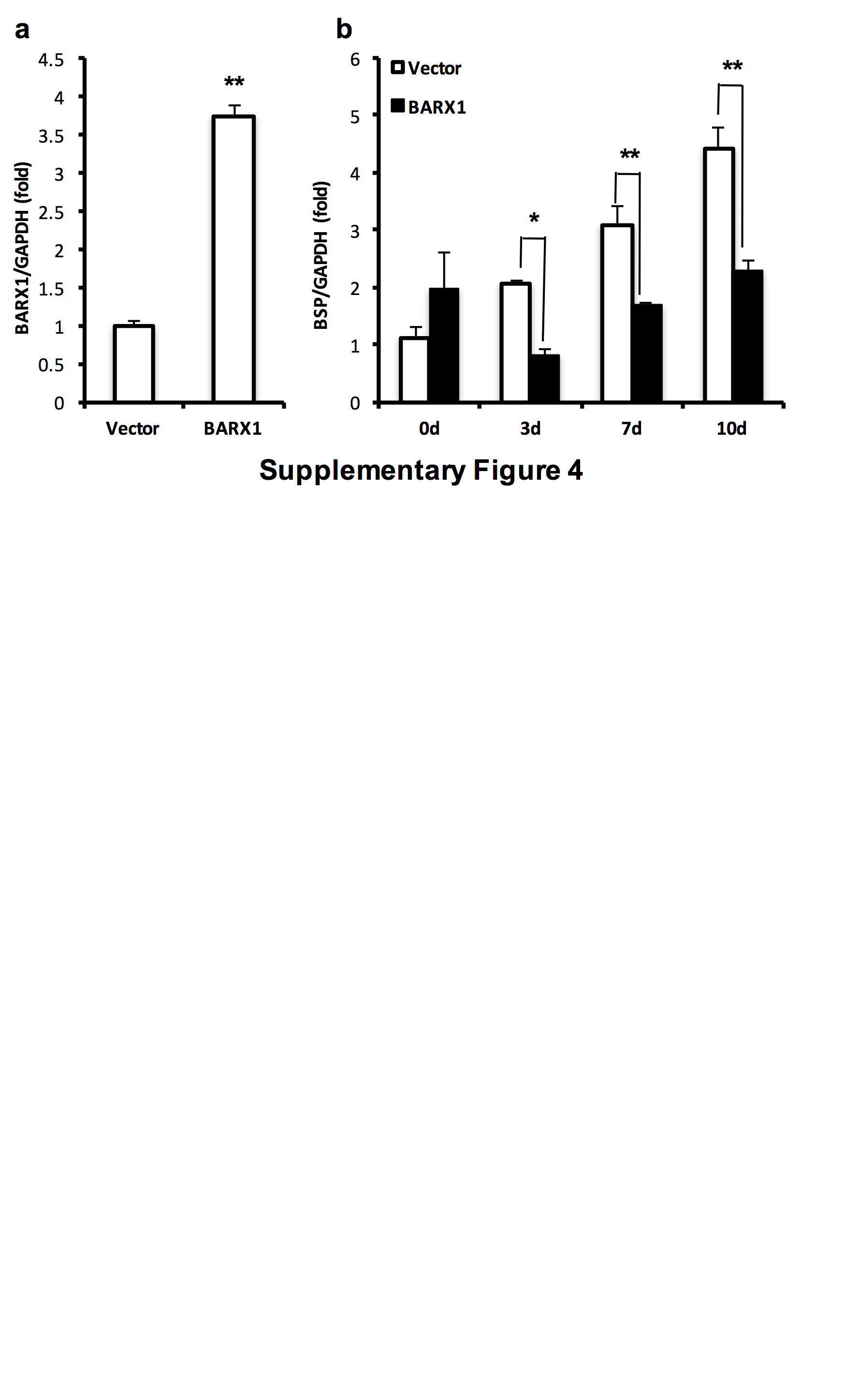

Supplement: Supplementary file 4 [file CPR-52-e12522-s004.tif]
