# Supplementary material for: AP2a enhanced the osteogenic differentiation of mesenchymal stem cells by inhibiting the formation of YAP/RUNX2 complex and BARX1 transcription
Source: Cell Prolif. 2018 Nov 15;52(1):e12522. doi: 10.1111/cpr.12522 (PMC6430486; doi:10.1111/cpr.12522)
Supplement: Supplementary file 5 [file CPR-52-e12522-s005.docx]

| Supplementary Table 1. Primers sequences used in the real-time RT-PCR | |
| --- | --- |
| Gene Symbol | Primer Sequences (5’-3’) |
| BARX1-F | CGCTTCGAGAAGCAGAAGTA |
| BARX1-R | CTTCATCCTCCGATTCTGGT |
| BSP-F | CAGGCCACGATATTATCTTTACA |
| BSP-R | CTCCTCTTCTTCCTCCTCCTC |
| YAP-F | CACAGCATGTTCGAGCTCAT |
| YAP-R | GACTACTCCAGTGGGGGTCA |
| GAPDH-F | CGGACCAATACGACCAAATCCG |
| GAPDH-R | AGCCACATCGCTCAGACACC |
