# Supplementary material for: AP2a enhanced the osteogenic differentiation of mesenchymal stem cells by inhibiting the formation of YAP/RUNX2 complex and BARX1 transcription
Source: Cell Prolif. 2018 Nov 15;52(1):e12522. doi: 10.1111/cpr.12522 (PMC6430486; doi:10.1111/cpr.12522)
Supplement: Supplementary file 6 [file CPR-52-e12522-s006.docx]

| Supplementary table 2. Primers sequences used in the real-time PCR for ChIP assays | |
| --- | --- |
| Gene Symbol | Primer Sequences (5’-3’) |
| BARX1-AP2a-BS1-F | GGAGAGACAGTGGGCTCTTG |
| BARX1-AP2a-BS1-R | GCCTGGAGTGAGGACAAAGA |
| BARX1-AP2a-BS2-F | CAGGTGCCAGGGACTGAG |
| BARX1-AP2a-BS2-R | AAGTCCTTTCTCCAGCTCCA |
| BARX1-AP2a-BS3-F | TCCCACCAGAGTTTGGTCTT |
| BARX1-AP2a-BS3-R | ACTGTTACTGGGCGGAGTTG |
| BARX1-AP2a-BS4-F | CTCTGTGCCCTCCGTCAC |
| BARX1-AP2a-BS4-R | AGACCAAAAGAGGCGTGAGA |
| BARX1-AP2a-BS5-F | CTCCTTTTTGGCCTCCCTTC |
| BARX1-AP2a-BS5-R | CCCAACCCCTAGGCTGAG |
| BARX1-AP2a-BS(5kb-down)-F | GGGATCTGACCAGCATCAGT |
| BARX1-AP2a-BS(5kb-down)-R | AAGATTCGCAAGGCAGCTAA |
