# Supplementary material for: AP2a enhanced the osteogenic differentiation of mesenchymal stem cells by inhibiting the formation of YAP/RUNX2 complex and BARX1 transcription
Source: Cell Prolif. 2018 Nov 15;52(1):e12522. doi: 10.1111/cpr.12522 (PMC6430486; doi:10.1111/cpr.12522)
Supplement: Supplementary file 7 [file CPR-52-e12522-s007.ppt]

## Slide 1
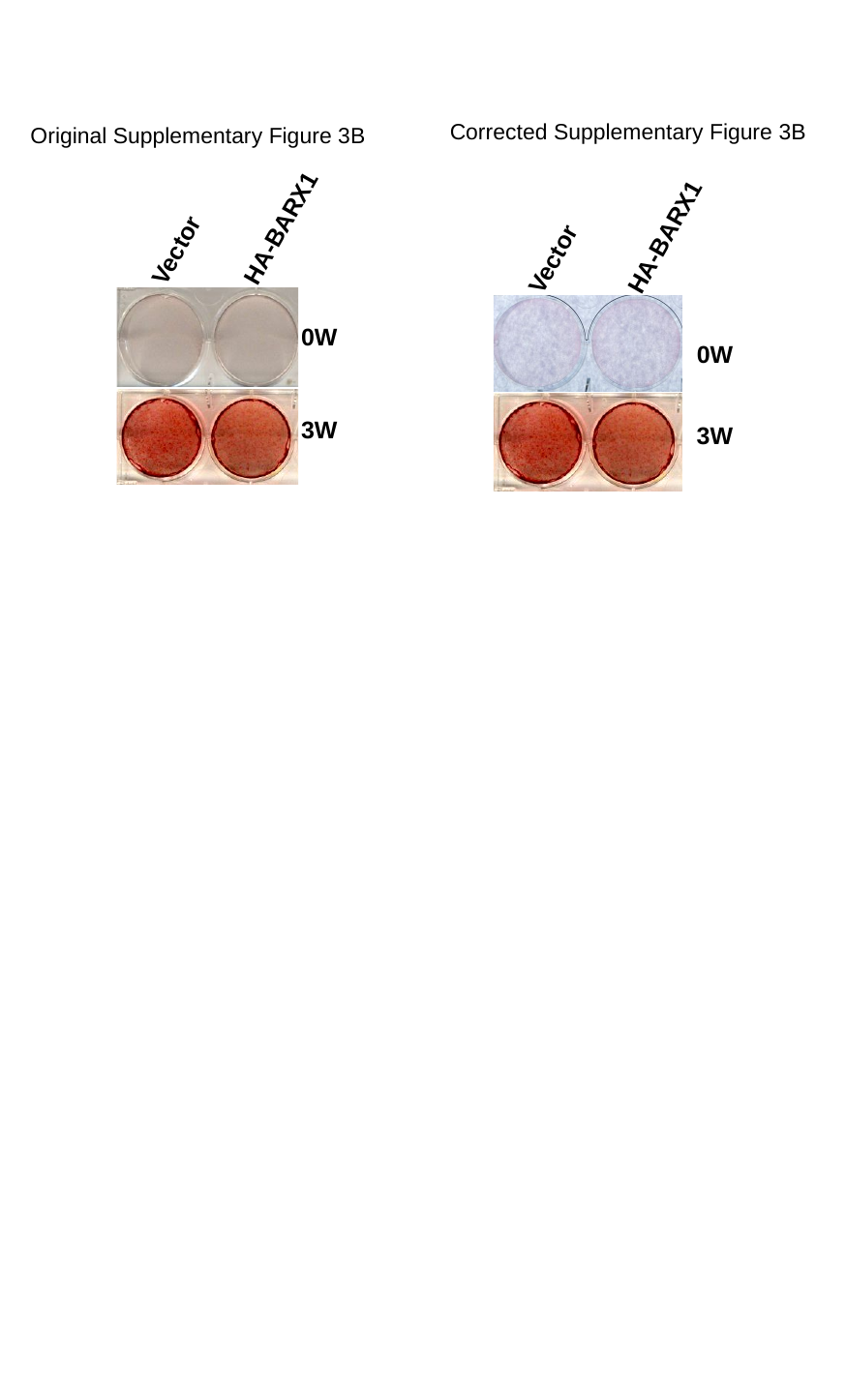

Corrected Supplementary Figure 3B
Original Supplementary Figure 3B
HA-BARX1
HA-BARX1
Vector
Vector
0W
0W
3W
3W
